# Evaluation of Routine Flexible Sigmoidoscopy After CT-Confirmed Acute Diverticulitis: A Retrospective Study

**DOI:** 10.7759/cureus.90691

**Published:** 2025-08-21

**Authors:** Hatim Albirnawi, Amr K Ebrahim, Arian Rahim, Mohamed Hassan, Aoff Khalil, Mahmud Riad, Syed Shah

**Affiliations:** 1 Upper GI Surgery, Maidstone and Tunbridge Wells NHS Trust, Maidstone, GBR; 2 Surgery, Maidstone and Tunbridge Wells NHS Trust, Maidstone, GBR; 3 General Surgery, Cairo University, Cairo, EGY; 4 General Surgery, Maidstone and Tunbridge Wells NHS Trust, Maidstone, GBR; 5 General Surgery, Heartlands Hospital, Birmingham, GBR; 6 Colorectal Surgery, Maidstone and Tunbridge Wells NHS Trust, Maidstone, GBR

**Keywords:** colonoscopy, colorectal cancer, computed tomography, diverticulitis, endoscopy, flexible sigmoidoscopy

## Abstract

Background: Acute diverticulitis is a common condition with increasing prevalence in Western countries. Current guidelines recommend endoscopic follow-up after an episode of acute diverticulitis to exclude malignancy, despite limited evidence supporting this practice. This study evaluates the utility of routine flexible sigmoidoscopy following CT-confirmed acute diverticulitis.

Methods: A retrospective cohort study was conducted at Maidstone and Tunbridge Wells NHS Trust from 2020 to 2022. We analysed 429 patients with CT-confirmed acute uncomplicated diverticulitis (Hinchey 1) who subsequently underwent flexible sigmoidoscopy six to eight weeks after initial presentation. Patient demographics, CT findings, and flexible sigmoidoscopy results were collected and analysed.

Results: Of the 429 patients included (180 males, 249 females; mean age 64 years), none were found to have colonic malignancy on follow-up flexible sigmoidoscopy. Only three patients (0.69%) had polyps: one inflammatory, one non-specific hyperplastic, and one tubular adenoma with low-grade dysplasia.

Conclusion: Our findings suggest that routine flexible sigmoidoscopy following CT-confirmed acute uncomplicated diverticulitis may not be necessary. Less invasive initial investigations, such as the faecal immunochemical test (FIT), could be considered for high-risk patients, potentially reducing unnecessary endoscopic procedures, associated patient risks, and healthcare costs.

## Introduction

In recent decades, there has been a notable increase in the prevalence of diverticular disease across the Western world [1,2]. Despite typically affecting the elderly population, diverticular disease is now increasingly affecting younger individuals, which has resulted in a greater number of investigations and hospital-related admissions [1]. It is estimated that 300,000 hospitalisations related to diverticular disease occur every year, costing healthcare services roughly £3 billion per annum [2].

Diverticulitis is typically classified into simple and uncomplicated, or complicated [3]. Complications of diverticulitis include bleeding, perforation, abscess, or bowel obstruction [3,4]. The preferred diagnostic modality is computed tomography (CT) [3,4]. In cases of complicated diverticulitis, or in patients with more concerning radiological findings, endoscopic follow-up is recommended after the acute phase in order to exclude neoplasms and inflammatory bowel disease, which may present similarly [5,6]. Additionally, flexible sigmoidoscopy is a cost-effective and safer procedure when compared to other forms of endoscopy, given that it requires fewer resources and is associated with a lower risk of bowel perforation [7].

There have been ongoing debates regarding the need for endoscopy following an episode of acute diverticulitis [8,9]. CT imaging allows for a high degree of sensitivity and specificity [4,10]; however, studies suggest that endoscopic evaluation would be beneficial in patients with a higher risk of colonic malignancy, such as a diagnosis of complicated diverticulitis, ongoing symptoms, and over 70 years of age [3,8].

This study aims to evaluate whether routine flexible sigmoidoscopy provides additional diagnostic yield in patients with CT-confirmed uncomplicated diverticulitis.

## Materials and methods

This single-centre, retrospective cohort study was conducted at Maidstone and Tunbridge Wells NHS Trust, a district general hospital serving a diverse population in Kent, England. The study was conducted from January 2020 to December 2022, encompassing a comprehensive review of patients presenting with acute diverticulitis during this timeframe [2].

Patient selection and inclusion criteria

We conducted a systematic electronic database search to identify patients who were initially diagnosed with acute diverticulitis based on CT imaging findings [3,4]. The inclusion criteria were as follows: (1) adult patients (≥18 years of age); (2) CT-confirmed diagnosis of acute uncomplicated diverticulitis (Hinchey Classification Grade 1) [4]; (3) conservative management during the acute episode; (4) subsequent follow-up with flexible sigmoidoscopy performed six to eight weeks after initial presentation [7]; and (5) complete medical records available for review.

To ensure study rigor and homogeneity of the patient cohort, the following exclusion criteria were applied: (1) patients who underwent colonoscopy instead of flexible sigmoidoscopy during follow-up [3]; (2) evidence of complicated diverticulitis (Hinchey Classification Grades 2-4) (including pericolic or pelvic abscess formation, generalized purulent peritonitis, generalized faecal peritonitis, and bowel perforation or obstruction [4]); (3) patients without CT-confirmed diagnosis of acute diverticulitis [3,4]; (4) those who have incomplete medical records or imaging studies; (5) patients who did not undergo endoscopic follow-up [2]; (6) who have previous history of colorectal malignancy [9]; (7) who have active inflammatory bowel diseases [5,6]; and (7) those lost to follow-up.

Data collection and variables

Patient identification was performed through a dual-search methodology. Initially, we searched the hospital's electronic patient record system for patients with a primary or secondary diagnosis of acute diverticulitis. A secondary search was conducted by reviewing electronic radiology reports to identify patients with CT-confirmed acute diverticulitis classified as Hinchey Grade 1 [4].

Demographic and clinical data collected included (1) patient age and gender; (2) CT imaging findings and radiological reports [3,4]; (3) Hinchey classification grade [4]; (4) management approach during an acute episode; (5) timing of follow-up endoscopy [7]; (6) flexible sigmoidoscopy findings and results [2,3]; (7) histopathological results of any biopsies taken; and (8) incidence of colonic malignancy or premalignant lesions [9-11].

Definition and assessment of red flag symptoms

Red flag symptoms were defined according to established clinical guidelines for colorectal cancer screening and were systematically assessed during the initial presentation and follow-up period [9,12]. The following primary red flag symptoms were specifically evaluated and documented: (1) rectal bleeding (visible blood in stool or on toilet paper); (2) unexplained weight loss (>5% of body weight over six months without intentional dieting); (3) iron deficiency anaemia (haemoglobin <12 g/dL in women, <13 g/dL in men with evidence of iron deficiency); (4) persistent altered bowel habit (change in stool frequency, consistency, or calibre lasting >6 weeks); and (5) new-onset constipation or diarrhoea in patients >50 years of age.

The following are the secondary risk factors: (1) age >50 years at presentation; (2) family history of colorectal cancer; (3) personal history of inflammatory bowel disease; and (4) previous adenomatous polyps.

Data collection methodology for red flag symptoms

Red flag symptoms were systematically collected through a standardized approach.

Electronic health record review: All patient records were reviewed for documented symptoms at initial presentation, during hospitalization, and at follow-up appointments.

Structured data extraction: A standardized data collection form was used to ensure consistent documentation of symptoms across all cases.

Validation process: All red flag symptom documentation was independently reviewed by two investigators to ensure accuracy and completeness of data collection.

Patients were included in the endoscopic follow-up protocol if they presented with any of the defined red flag symptoms or met the age criterion (>50 years), in accordance with current clinical guidelines for post-diverticulitis surveillance [9-11,13].

Ethical considerations

All patient data were anonymized and de-identified prior to analysis. No personally identifiable information was retained by the research team or included in this study. Ethical approval was obtained from the local hospital audit committee in accordance with institutional guidelines for retrospective clinical research.

Statistical analysis

Descriptive statistics were used to summarize patient demographics and clinical outcomes. Categorical variables were expressed as frequencies and percentages, while continuous variables were presented as means with ranges where appropriate.

Flexible sigmoidoscopy technique

All flexible sigmoidoscopy procedures were performed by experienced endoscopists following standard protocols [12]. Patients underwent bowel preparation with phosphate enemas administered one to two hours prior to the procedure. The procedures were performed with the patient in the left lateral position using standard flexible sigmoidoscopes. The scope was advanced to the descending colon or splenic flexure as tolerated by the patient. During withdrawal, careful mucosal inspection was performed with adequate insufflation. Any abnormal findings, including diverticulosis, inflammation, polyps, or suspicious lesions, were documented. Biopsies were taken of any suspicious areas or polyps encountered during the procedure. All procedures were performed six to eight weeks after the initial episode of acute diverticulitis to allow for adequate mucosal healing.

## Results

Patient demographics and clinical characteristics

A total of 456 patients were identified with CT-confirmed acute diverticulitis during the study period (2020-2022). Of these, 429 patients (94.1%) underwent flexible sigmoidoscopy during follow-up and were included in our analysis, while 26 patients (5.7%) had colonoscopy and were excluded. One patient (0.2%) was lost to follow-up.

The study cohort (n=429) consisted of 249 females (58.0%) and 180 males (42.0%), with a male-to-female ratio of 1:1.38 (Table 1). The mean age was 64 years, with a range of 26-96 years. The age distribution analysis revealed that 312 patients (72.7%) were over 50 years of age, while 117 patients (27.3%) were under 50 years. The majority of patients (n=287, 66.9%) presented with left-sided diverticulitis, while 142 patients (33.1%) within our cohort had right-sided diverticulitis.

**Table 1:** Patient demographics and clinical characteristics.

Characteristic	Value
Gender	
Female	249 (58.0%)
Male	180 (42.0%)
Age (Years)	
Mean ± SD	64 ± 15.3
Range	26-96
Age Distribution	
<50 years	117 (27.3%)
50-69 years	156 (36.4%)
≥70 years	156 (36.4%)
Diverticulitis Location	
Left-sided	287 (66.9%)
Right-sided/Bilateral	142 (33.1%)
Comorbidities	
Hypertension	187 (43.6%)
Diabetes mellitus	92 (21.4%)
Coronary artery disease	68 (15.9%)
Chronic kidney disease	41 (9.6%)
Inflammatory bowel disease	0 (0%)

CT findings

All patients included in the study had CT-confirmed acute uncomplicated diverticulitis (Hinchey 1) [11]. The most common CT findings included bowel wall thickening (n=398, 92.8%), pericolic fat stranding (n=412, 96.0%), and presence of diverticula (n=429, 100%) [4,10]. No patients in our cohort had evidence of perforation, abscess formation, or fistula on initial CT imaging, as these would have classified them as complicated diverticulitis and excluded them from the study.

**Figure 1:**
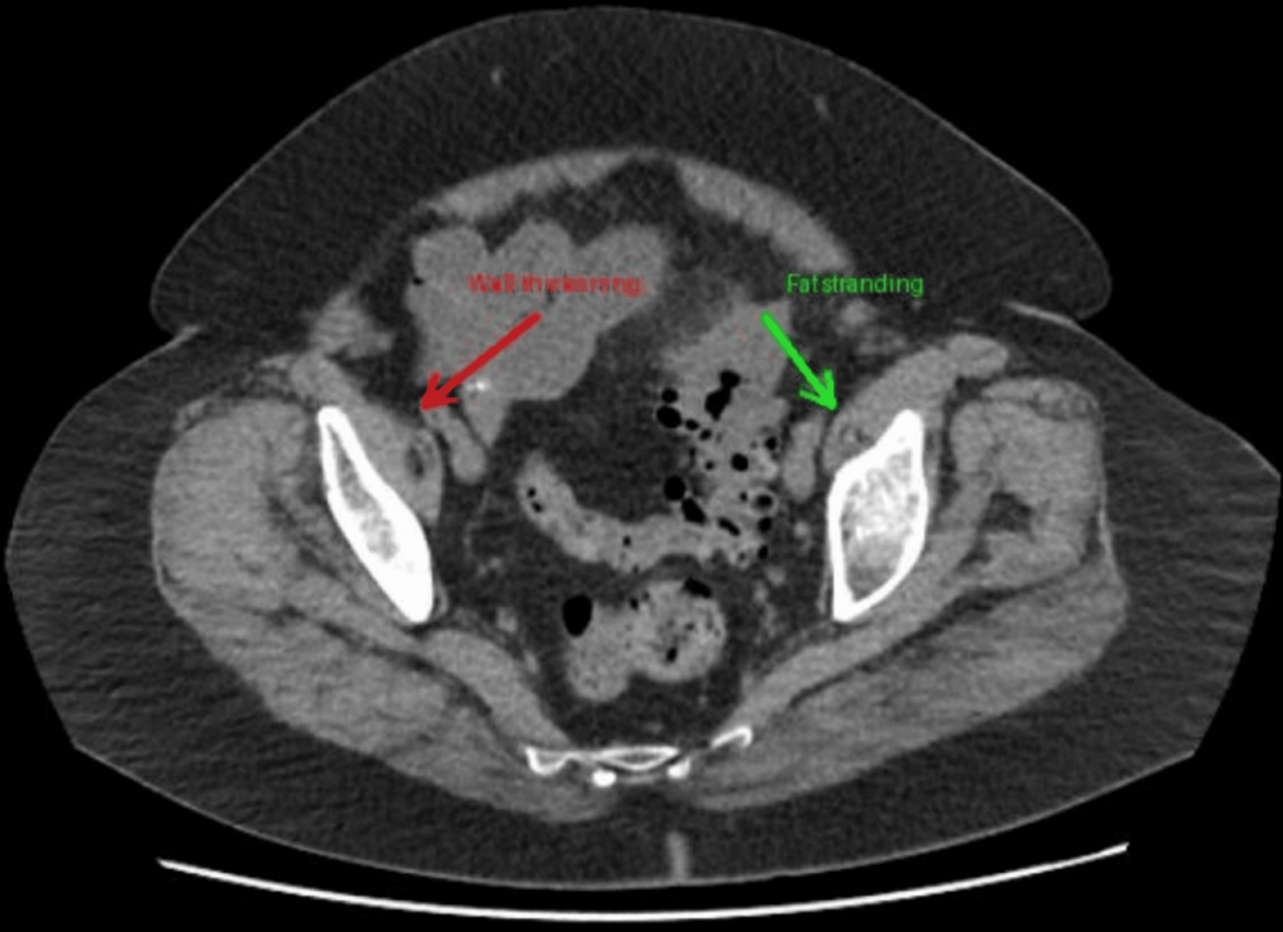
CT scan showing acute diverticulitis with characteristic bowel wall thickening and pericolic fat stranding. An axial CT scan showing acute diverticulitis with characteristic bowel wall thickening (red arrow) and pericolic fat stranding (green arrow). Original image from an anonymized patient in this study cohort. Image Credits: Maidstone and Tunbridge Wells NHS Trust, used with permission (relevant findings discussed in [4,10,11]).

Figure 1 presents the CT scan showing acute diverticulitis with characteristic bowel wall thickening and pericolic fat stranding.

Flexible sigmoidoscopy findings

All 429 patients underwent flexible sigmoidoscopy at a median of seven weeks (range: 6-9 weeks) after the initial episode of acute diverticulitis [5,6]. The procedures were well-tolerated, with no reported complications such as perforation or significant bleeding [7].

**Figure 2:**
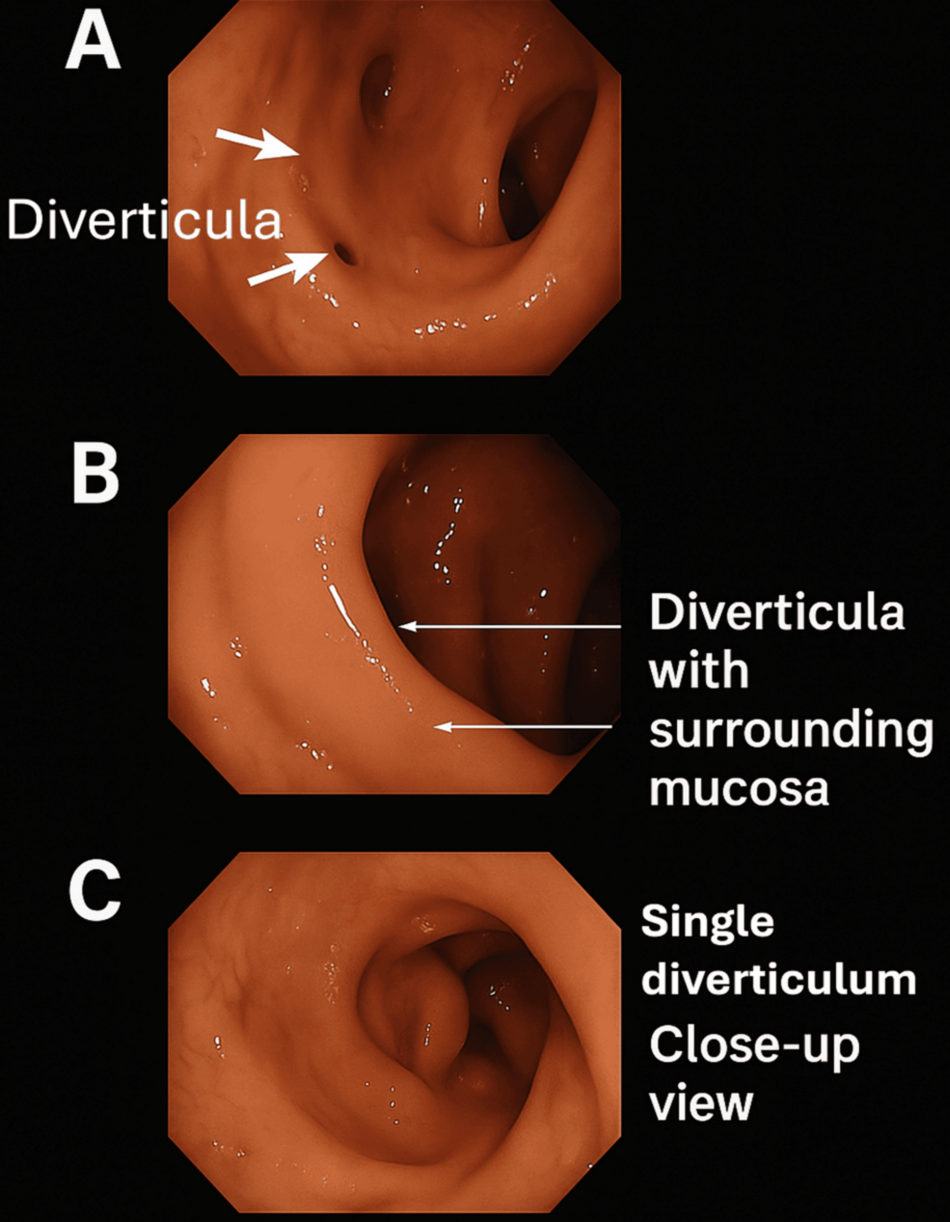
Flexible sigmoidoscopy showing diverticula in the sigmoid colon. Flexible sigmoidoscopy images. (A) View of diverticula in the sigmoid colon. (B) Another view of diverticula with surrounding mucosa. (C) Close-up view of a single diverticulum. Original image from an anonymized patient in this study cohort. Image Credits: Maidstone and Tunbridge Wells NHS Trust, used with permission (endoscopic findings discussed in [7,12]).

 Figure 2 presents the flexible sigmoidoscopy images (A, B, C) showing various views of diverticula in the sigmoid colon.

The endoscopic findings are summarized in Table 2. Diverticulosis was confirmed in all 429 patients (100%). Signs of recent inflammation were observed in 87 patients (20.3%), characterized by mucosal erythema and oedema. Only three patients (0.69%) had polyps identified during the procedure: one inflammatory polyp (0.23%), one non-specific hyperplastic polyp (0.23%), and one tubular adenoma with low-grade dysplasia (0.23%). Importantly, no cases of colonic malignancy were detected in any of the 429 patients.

**Table 2:** Flexible sigmoidoscopy findings.

Finding	Number of Patients (%)
Diverticulosis	429 (100%)
Signs of recent inflammation	87 (20.3%)
Polyps	3 (0.69%)
Inflammatory	1 (0.23%)
Non-specific hyperplastic	1 (0.23%)
Tubular adenoma with low-grade dysplasia	1 (0.23%)
Colonic malignancy	0 (0%)
Other findings	23 (5.4%)

Polyp characteristics and management

All three polyps were small (≤5 mm) and were completely removed during the flexible sigmoidoscopy procedure. The tubular adenoma with low-grade dysplasia was found in a 72-year-old male patient with no family history of colorectal cancer. This patient was recommended for surveillance colonoscopy in three years according to standard polyp surveillance guidelines [13]. The inflammatory and hyperplastic polyps were considered benign with no further follow-up required beyond standard care.

Comparison of patient subgroups

We performed a subgroup analysis comparing patients with and without red flag symptoms (Table 3). Among the 429 patients, 187 (43.6%) presented with at least one red flag symptom (rectal bleeding, unexplained weight loss, iron deficiency anaemia, or altered bowel habit) [2,13], while 242 (56.4%) had no red flag symptoms. Notably, the incidence of polyps was not significantly different between these groups (0.5% vs. 0.8%, p=0.74), and no malignancy was detected in either group.

**Table 3:** Comparison of patients with and without red flag symptoms. Statistical tests: Chi-square test used for categorical variables; Independent t-test used for continuous variables. 95% confidence intervals calculated using exact binomial method (Clopper-Pearson) for proportions and t-distribution for means.

Characteristic	With Red Flag Symptoms (n=187)	Without Red Flag Symptoms (n=242)	Test Statistic	p-value
Gender				
Female	112 (59.9%, 95% CI: 52.6-66.9%)	137 (56.6%, 95% CI: 50.2-62.9%)	χ² = 0.47	0.49
Male	75 (40.1%, 95% CI: 33.1-47.4%)	105 (43.4%, 95% CI: 37.1-49.8%)		
Age (years)				
Mean ± SD	66.2 ± 14.8 (95% CI: 64.1-68.3)	62.4 ± 15.5 (95% CI: 60.4-64.4)	t = 2.58	0.01
Findings				
Polyps	1 (0.5%, 95% CI: 0.01-2.9%)	2 (0.8%, 95% CI: 0.1-2.9%)	χ² = 0.11	0.74
Malignancy	0 (0%, 95% CI: 0-2.0%)	0 (0%, 95% CI: 0-1.5%)	N/A	N/A

Additionally, we analysed the outcomes based on age groups (Table 4).

**Table 4:** Comparison of the findings by age group. Statistical tests: Chi-square test used for overall polyp comparison; Fisher’s exact test used for individual polyp types due to small cell counts. 95% confidence intervals calculated using exact binomial method (Clopper-Pearson) for proportions.

Finding	Age <70 years (n=273)	Age ≥70 years (n=156)	Test Statistic	p-value
Polyps	2 (0.73%, 95% CI: 0.09-2.6%)	1 (0.64%, 95% CI: 0.02-3.5%)	χ² = 0.01	0.91
Inflammatory	1 (0.37%, 95% CI: 0.01-2.0%)	0 (0%, 95% CI: 0-2.3%)	Fisher’s exact	1.00
Hyperplastic	1 (0.37%, 95% CI: 0.01-2.0%)	0 (0%, 95% CI: 0-2.3%)	Fisher’s exact	1.00
Tubular adenoma	0 (0%, 95% CI: 0-1.4%)	1 (0.64%, 95% CI: 0.02-3.5%)	Fisher’s exact	0.36
Malignancy	0 (0%, 95% CI: 0-1.4%)	0 (0%, 95% CI: 0-2.3%)	N/A	N/A

Among patients aged ≥70 years (n=156), only one polyp (0.64%) was detected (the tubular adenoma), while in patients <70 years (n=273), two polyps (0.73%) were found (the inflammatory and hyperplastic polyps). Again, no malignancy was detected in either age group.

Cost analysis

We conducted a basic cost analysis of routine flexible sigmoidoscopy following acute diverticulitis [14]. Based on the average cost of a flexible sigmoidoscopy procedure (approximately £450 per procedure), the total expenditure for the 429 procedures was estimated at £193,050. Given that no cases of malignancy were detected and only three benign polyps were found, the cost per clinically significant finding (defined as cancer or advanced adenoma) was extremely high at £193,050 per case of advanced adenoma, with no cases of cancer detected.

## Discussion

The debate regarding the necessity of endoscopic follow-up after an episode of acute diverticulitis has been ongoing in the medical community [8-10]. Our study contributes valuable evidence to this discussion by demonstrating that routine flexible sigmoidoscopy following CT-confirmed acute uncomplicated diverticulitis resulted in no cases of colorectal malignancy among 429 patients.

Comparison with previous studies

Our findings align with several recent studies questioning the value of routine endoscopic evaluation after acute diverticulitis. Sharma et al. conducted a systematic review and meta-analysis of 11 studies involving 1,970 patients and found a pooled incidence of colorectal cancer of only 1.6% following an episode of acute diverticulitis [8]. However, when they restricted their analysis to patients with uncomplicated diverticulitis, as in our study population, the risk of colorectal cancer dropped to 0.7%, which is comparable to the risk in the general population [8].

Similarly, Hannan et al. reported no cases of malignancy among 271 patients who underwent follow-up endoscopy after acute sigmoid diverticulitis [2]. Their conclusion that sigmoidoscopy might be sufficient rather than full colonoscopy is supported by our findings, though our results further suggest that even sigmoidoscopy may be unnecessary in uncomplicated cases.

Abdulazeez et al. compared outcomes between patients who underwent flexible sigmoidoscopy versus colonoscopy after left-sided diverticulitis and found no significant difference in diagnostic yield for colorectal cancer between the two approaches [3]. Our study extends these findings by questioning whether any form of endoscopic follow-up is necessary for uncomplicated cases. In contrast to our findings, some older studies have reported higher rates of colorectal cancer detection following acute diverticulitis. For instance, Lau et al. reported a 2.1% incidence of colorectal cancer in patients with acute diverticulitis [15]. However, their study included both complicated and uncomplicated cases and was conducted before the widespread use of high-resolution CT imaging, which has significantly improved diagnostic accuracy [4,10].

Current guidelines and our findings

Despite the growing evidence questioning routine endoscopy after uncomplicated diverticulitis, several professional organizations continue to recommend follow-up endoscopy. The Association of Coloproctology of Great Britain and Ireland (ACPGBI) guidelines [9], the American Society of Colon and Rectal Surgeons [10], and the American College of Gastroenterology [11] all recommend endoscopic evaluation after an episode of acute diverticulitis.

These recommendations are primarily based on the theoretical concern that the initial CT scan might miss a malignancy that presents similarly to diverticulitis [10,15]. However, our study, along with other recent evidence, suggests that modern CT imaging is highly accurate in differentiating between diverticulitis and colorectal cancer, particularly in uncomplicated cases [4,10].

Subgroup analysis and risk stratification

Our subgroup analysis comparing patients with and without red flag symptoms revealed no significant difference in the detection of polyps or malignancy between these groups. This finding warrants reconsideration of red flag-driven endoscopy referral practices in guidelines [2,13].

Similarly, our age-based analysis showed no significant difference in the detection of clinically significant lesions between patients under and over 70 years of age. This suggests that age alone may not be a reliable criterion for determining the need for follow-up endoscopy after uncomplicated diverticulitis [3,8].

These findings support a more targeted approach to follow-up, focusing on patients with complicated diverticulitis or those with persistent symptoms despite resolution of the acute episode, rather than subjecting all patients to invasive procedures [8,12].

Cost-effectiveness considerations

The cost analysis presented in our results section highlights the significant financial implications of routine endoscopic follow-up. With an estimated cost of £193,050 for the 429 procedures and only three benign polyps detected (none of which were advanced adenomas), the cost-effectiveness of this approach is questionable [14].

Laurie et al. conducted a multi-centre review assessing the role of routine colonoscopy after acute diverticulitis, contributing to the ongoing debate about its necessity [14]. Our findings further support the notion that a more selective approach to endoscopic follow-up, focusing on higher-risk populations, could lead to a more efficient allocation of healthcare resources.

Alternative follow-up strategies

Given the low yield of flexible sigmoidoscopy in our study, alternative follow-up strategies deserve consideration. The faecal immunochemical test (FIT) has shown promise as a non-invasive screening tool for colorectal cancer [9,16]. The ACPGBI guidelines note that FIT testing has resulted in the identification of more colorectal cancers and advanced adenomas compared to traditional approaches [9].

According to the joint guideline from the ACPGBI and the British Society of Gastroenterology (BSG), the FIT demonstrates high diagnostic accuracy in symptomatic patients, including those with diverticular disease, and can help prioritize colonoscopy referrals [16].

Strengths and limitations

Our study has several strengths, including its relatively large sample size, the homogeneity of the study population (all with CT-confirmed uncomplicated diverticulitis), and the standardized timing of flexible sigmoidoscopy (six to eight weeks after the acute episode).

However, we acknowledge several limitations. First is the selection bias: this was a retrospective study conducted at a single centre. There may be inherent selection bias in determining which patients underwent flexible sigmoidoscopy versus colonoscopy during follow-up. The decision-making process for endoscopic modality selection was not standardized and may have been influenced by physician preference, patient factors, or clinical characteristics not captured in our analysis. This could potentially affect the generalizability of our findings, as patients selected for sigmoidoscopy may represent a lower-risk subset compared to those who underwent colonoscopy.

Second is that we did not have a control group of patients who did not undergo flexible sigmoidoscopy, which would have allowed for a direct comparison of outcomes. Third is the limited follow-up period: Our study assessed endoscopic findings only at six to eight weeks post-acute diverticulitis episode and did not evaluate long-term colorectal cancer risk beyond this initial follow-up period. Malignancies may develop or become detectable months to years after the initial presentation, and our short-term follow-up may have missed delayed cancer diagnoses. A longer surveillance period would be necessary to comprehensively assess the true cancer detection yield of routine endoscopy. However, given the natural history of adenoma-to-carcinoma progression [13], it is unlikely that a significant number of cancers would have been missed due to the timing of our endoscopic evaluation.

Fourth, our cost analysis focused solely on direct healthcare costs related to endoscopic procedures and did not account for indirect costs, including patient time off work, transportation expenses, procedure-related complications, or the psychological burden and discomfort experienced by patients undergoing endoscopy. These indirect costs may represent a substantial economic impact that warrants consideration in future comprehensive cost-effectiveness analyses.

Clinical implications

The findings of our study have important clinical implications for the management of patients with acute diverticulitis. If routine flexible sigmoidoscopy after uncomplicated diverticulitis does not yield significant findings, as our results suggest, then healthcare resources could be redirected to more effective interventions [14].

A more selective approach to endoscopic follow-up, focusing on patients with complicated diverticulitis, persistent symptoms, or positive FIT results [9,16], could reduce unnecessary procedures, decrease healthcare costs, and improve patient experience without compromising clinical outcomes.

Future research directions

Future research should focus on prospective studies comparing different follow-up strategies after acute diverticulitis, including no endoscopic follow-up, selective endoscopy based on risk factors, and non-invasive testing such as FIT [16]. Additionally, longer-term follow-up studies would be valuable to assess the natural history of patients with acute diverticulitis who do not undergo endoscopic evaluation.

Cost-effectiveness analyses incorporating quality of life measures would also provide valuable information for healthcare policy decisions regarding the optimal management of these patients [14].

## Conclusions

This retrospective study of 429 patients with CT-confirmed acute uncomplicated diverticulitis found no cases of colorectal malignancy on follow-up flexible sigmoidoscopy, with only three benign polyps detected (0.69%). These findings strongly suggest that routine flexible sigmoidoscopy following CT-confirmed acute uncomplicated diverticulitis may be reconsidered in low-risk populations, as it represents a significant burden on healthcare resources without proportional clinical benefit. The absence of malignancy in our cohort, including among patients with red flag symptoms and those over 70 years of age, challenges current practice guidelines that recommend routine endoscopic follow-up. Our cost analysis further demonstrates the questionable economic value of this approach, with substantial expenditure yielding minimal clinically significant findings. We propose that a more selective approach to endoscopic evaluation be considered, potentially incorporating non-invasive screening methods such as the FIT as an initial triage tool. This strategy could significantly reduce unnecessary invasive procedures, decrease healthcare costs, and improve patient experience while maintaining high standards of clinical care. Future prospective studies comparing different follow-up strategies with longer-term outcomes would further refine optimal management protocols for patients with acute diverticulitis.
